# Biomarkers of angiogenesis and their role in the development of VEGF inhibitors

**DOI:** 10.1038/sj.bjc.6605483

**Published:** 2009-12-15

**Authors:** N Murukesh, C Dive, G C Jayson

**Affiliations:** 1 Department of Medical Oncology, Manchester Academic Health Science Centre, The Christie NHS Foundation Trust, Cancer Research UK and University of Manchester, Wilmslow Road, Withington, Manchester M20 4BX, UK; 2 Cancer Research UK and Clinical and Experimental Pharmacology Group, Manchester Cancer Research Centre, Paterson Institute of Cancer Research, Withington, Manchester M20 4BX, UK

**Keywords:** VEGF, angiogenesis, biomarkers

## Abstract

Vascular endothelial growth factor (VEGF) has been confirmed as an important therapeutic target in randomised clinical trials in multiple disease settings. However, the extent to which individual patients benefit from VEGF inhibitors is unclear. If we are to optimise the use of these drugs or develop combination regimens that build on this efficacy, it is critical to identify those patients who are likely to benefit, particularly as these agents can be toxic and are expensive. To this end, biomarkers have been evaluated in tissue, in circulation and by imaging. Consistent drug-induced increases in plasma VEGF-A and blood pressure, as well as reductions in soluble VEGF-R2 and dynamic contrast-enhanced MRI parameters have been reported. In some clinical trials, biomarker changes were statistically significant and associated with clinical end points, but there is considerable heterogeneity between studies that are to some extent attributable to methodological issues. On the basis of observations with these biomarkers, it is now appropriate to conduct detailed prospective studies to define a suite of predictive, pharmacodynamic and surrogate response biomarkers that identify those patients most likely to benefit from and monitor their response to this novel class of drugs.

Angiogenesis, the process of new blood vessel formation, is critical for the growth and metastasis of tumours. Early in tumourigenesis, an ‘angiogenic switch’ is activated by hypoxia, activated oncogenes and/or metabolic stress. The previously closely maintained physiological balance that keeps adult vasculature in a relatively quiescent state is then tipped in favour of angiogenesis through the expression of pro-angiogenic growth factors, such as vascular endothelial growth factor (VEGF) (Hanahan and Folkman, 1996). Vascular endothelial growth factor has been confirmed in multiple clinical trials as an important target for solid tumour treatment, but it is not clear who benefits from this class of drugs; this is an issue of increasing importance, bearing in mind the toxicity and expense of VEGF inhibitors in addition to the need to generate combination regimens that include VEGF inhibitors.

Although several biomarkers associated with angiogenesis measured before treatment have shown to provide prognostic value, and a few biomarkers are pharmacodynamic, there is minimal information on biomarkers that are true surrogates for clinical response to VEGF inhibitors. As VEGF-targeted therapies progress through clinical development pipelines, it may take several years to determine their clinical efficacy and overall response data. Thus, there is a pressing need for predictive and pharmacodynamic biomarkers and for those that are true surrogates of clinical response.

## Vascular endothelial growth factor

Vascular endothelial growth factor is a homodimeric glycoprotein with a molecular weight of 45 kDa. The VEGF family includes VEGF-A (usually referred to as VEGF), VEGF-B, VEGF-C, VEGF-D and a structurally related molecule, placental growth factor (PlGF). Through alternative splicing of VEGF mRNA, 12 isoforms of VEGF have been identified (Nowak *et al*, 2008), most of which can activate the signal transducing receptors. However, through alternative splicing of exon 8, anti-angiogenic variants, designated as VEGF-A_xxxb_, can be formed, where xxx denotes the number of amino acids in the mature protein. Although present in the malignant colonic epithelium, the inhibitory effect of VEGF-A_165b_ is overcome by an excess of VEGF-A_165_, a relationship that contributes to the relative efficacy *in vivo* of anti-VEGF antibodies that bind both isoforms (Bates *et al*, 2002; Varey *et al*, 2008). The clinical significance of the ratio of activating and inactivating isoforms of VEGF to the activity of VEGF inhibitors remains unclear.

Three high-affinity VEGF tyrosine kinase receptors have been identified: VEGF receptor (VEGFR)-1 (flt-1), VEGFR-2 (flt-1/KDR) and VEGFR-3 (flt-4). The binding of VEGF to these receptors initiates a cascade of signalling pathways that mediate endothelial cell (EC) migration, proliferation, survival and permeability. Additional co-receptors include neuropilins that have traditionally been implicated in cell guidance (Kawasaki *et al*, 1999) and increased binding of VEGF to its signalling receptor (Soker *et al*, 1998). However, recent data have suggested that NRP-1 may regulate EC function independently of VEGFR-2 (Murga *et al*, 2005), and that VEGF_121_ can directly interact with NRP-1 without forming an NRP-1–VEGFR-2 complex (Pan *et al*, 2007).

Vascular endothelial growth factor-A interacts with both VEGFR-1 and VEGFR-2 to mediate angiogenesis, whereas VEGF-B and PlGF have high affinity for only VEGFR-1. Vascular endothelial growth factor-C and VEGF-D bind both VEGFR-2 and VEGFR-3 (Joukov *et al*, 1996; Achen *et al*, 1998) to regulate angiogenesis and have been implicated in lymphangiogenesis (Shibuya and Claesson-Welsh, 2006). Vascular endothelial growth factor receptor-2 is the principal receptor that promotes the pro-angiogenic action of VEGF-A and has been the principal target of anti-angiogenic therapies, although additional studies have underlined the importance of signalling through VEGFR-1 (Carmeliet *et al*, 2001).

Various strategies for inhibiting VEGF have been investigated over the last decade. These include neutralising antibodies to VEGF (Hurwitz *et al*, 2004), low-molecular-weight VEGFR tyrosine kinase inhibitors (TKis) (Motzer *et al*, 2006; Llovet *et al*, 2008) and soluble VEGFR constructs (VEGF-Trap) (Riely and Miller, 2007) (Table 1).

Angiogenesis and VEGF have been confirmed as targets of anti-cancer therapeutics in multiple disease settings. Randomised clinical trials in the first- and second-line treatment of metastatic colorectal cancer (Hurwitz *et al*, 2004; Giantonio *et al*, 2007), breast cancer (Miller *et al*, 2007), non-small-cell lung cancer (Sandler *et al*, 2006), renal cancer (Motzer *et al*, 2007) and hepatocellular carcinoma (Llovet *et al*, 2008) have demonstrated an improvement in response, progression-free survival (PFS) and/or overall survival (OS) when conventional therapy was supplemented by VEGF inhibitors.

The demonstration of a survival advantage conferred by VEGF inhibitors is of great importance. However, the initial promise of anti-angiogenic agents, namely the reduced prevalence of drug resistance and durable stabilisation of disease, has not been realised. Vascular endothelial growth factor inhibitors also have a range of host toxicities. They are expensive and optimal development of combination anti-vascular regimens requires the identification of those patients most likely to benefit from treatment with this class of drugs. Despite attempts, this goal has eluded us. In this study, we review the use of candidate predictive and/or pharmacodynamic biomarkers pertinent to VEGF inhibition and highlight approaches that are yet to be investigated.

## Biomarkers

A biomarker is a characteristic that is objectively measured and evaluated as an indicator of a normal biological process, a pathogenic process or of pharmacological response to a therapeutic intervention (Atkinson *et al*, 2001). Several categories of biomarkers have been described that are pertinent to cancer, namely screening, diagnostic, prognostic, predictive, pharmacological (pharmacodynamic, proof of mechanism and of concept), surrogate response and safety biomarkers. Biomarker assays need to be carefully validated and be robust, reliable and reproducible when applied in clinical contexts. As VEGF inhibitors are already licensed, the most important question to be addressed using biomarkers is who should be treated with this class of agent; that is, at this point in the development of VEGF inhibitors, there is a clear need to identify predictive, pharmacological and surrogate response biomarkers, and in particular, to discriminate between these and prognostic biomarkers (which inform on the progression of disease irrespective of treatment).

A number of confounding issues recur in the literature regarding the use of biomarkers: Are there enough samples from sufficiently large trials to detect a statistically significant result? Have assays been performed within each patient before drug administration to determine whether a change in a biomarker can be ascribed to the drug, that is, is baseline variation characterised? Have biomarker studies been carried out according to the standards of Good Clinical Laboratory Practice required by the EU clinical trial directive (2001/20/EC). Such issues are of greater than theoretical importance, as, for example, we know that inappropriate blood sample handling can lead to platelet activation and *ex vivo* release of PDGF and VEGF. Therefore, debate is ongoing regarding the optimal choice of specimen for the measurement of these biomarkers. Serum seems to be a popular choice; however, the release of the above factors during clotting can influence the values measured. However, considering the low sensitivity of ELISAs to detect plasma levels and the proposed scavenging of VEGF by platelets (George *et al*, 2000), serum levels might represent the truer picture. In this study, we discuss trials that have incorporated circulating molecular and cellular, tissue, genetic and/or imaging biomarkers that are related to VEGF and its inhibition. Hypertension is one of the most common toxicities in patients having VEGF inhibitors and, in this study, we also examine the differential benefits seen in patients experiencing hypertension.

## Circulating candidate biomarkers of angiogenesis

The majority of clinical trials that have evaluated VEGF inhibitors have involved investigation of either anti-VEGF antibodies or VEGFR TKis. The prototypic VEGF inhibitor is the monoclonal anti-VEGF antibody, bevacizumab. Table 2a summarises the data collected from studies of circulating biomarkers in cancer patients treated with bevacizumab and other antibody-based therapeutics. One of the first biomarkers to be evaluated was the plasma concentration of VEGF-A. However, of multiple trials, only the E4599 trial in non-small-cell lung cancer reported that the pre-treatment plasma concentration of VEGF was of prognostic significance (Dowlati *et al*, 2008). Intuitively, one would predict that the pre-treatment plasma concentration of VEGF would be most helpful in diseases that respond to single-agent VEGF inhibitors (e.g., renal, ovarian and hepatic cancer). However, the extent to which we can interpret such data is limited by, for example, studies that have been too small (Siegel *et al*, 2008) or in cases in which the limit of quantitation of ELISA was at a concentration that precluded interpretation of a significant proportion of biomarker data (Yang *et al*, 2003).

The initial phase I trials of anti-VEGF antibodies demonstrated that there was a logarithmic increase in the total plasma concentration of VEGF after drug administration (Gordon *et al*, 2001; Jayson *et al*, 2005). The source of this cytokine is not clear, but could reflect extensive loading of the extracellular matrix with VEGF in patients with advanced cancer. Thus, one hypothesis would be that the magnitude of the anti-VEGF antibody-induced change in plasma VEGF concentration might predict patient benefit. However, despite an interesting report in one small study (Siegel *et al*, 2008), this has not been confirmed in other trials, many of which were also compromised by their insufficient size. The other possibility is that anti-VEGF antibodies form inert complexes, causing a false increase in circulating VEGF levels. One study conducted using immunodepleted plasma has supported this assumption by showing a significant decrease in VEGF levels after treatment with bevacizumab (Loupakis *et al*, 2007).

The increase in plasma VEGF concentration in patients treated with anti-VEGF antibodies has also been seen in those receiving low-molecular-weight VEGFR TKis (Table 2b). A VEGFR TKi biomarker signature has emerged, in which the drugs induce an increase in plasma VEGF and PlGF, as well as reductions in soluble VEGFR-2 and VEGFR-3. Presumably, this biomarker signature reflects the larger repertoire of receptors targeted by VEGFR TKis compared with anti-VEGF antibodies. If true, one might not expect to see an increase in VEGFR-3 concentrations in patients receiving bevacizumab, although this has not been formally reported.

Although a principal aim of biomarker studies in patients receiving VEGF inhibitors is to identify those patients who are most likely to benefit, it is equally important to detect the onset of drug resistance and ideally the factors mediating this resistance, which is an area of increasing importance, given recent data that indicate the potential value of continuing treatment with VEGF inhibitors until disease progression (Hurwitz *et al*, 2004; Saltz *et al*, 2008). To date, few biomarker studies have identified clinically tractable mediators of resistance, but some recent data highlighted FGF-2 and SDF-1*α* as potential targets (Batchelor *et al*, 2007).

Circulating ECs (CECs) are believed to arise from vessel walls, either of mature vessels or the tumour vasculature. A subset of them is thought to originate from the bone marrow and represents circulating endothelial progenitor cells (CEPCs) (Lin *et al*, 2000). Normal adults have 1–20 CECs per ml in their peripheral blood, and the levels are shown to increase significantly in patients with advanced cancer (Rowand *et al*, 2007). After successful treatment, their concentration tends to normalise (Willett *et al*, 2005), in contrast to the situation in progressive disease (Beerepoot *et al*, 2004). In patients with breast cancer, one study demonstrated that the pre-treatment high concentration of CECs was a good prognostic factor (Dellapasqua *et al*, 2008), whereas another showed that in patients receiving metronomic doses of cytotoxic chemotherapy, an increase in circulating apoptotic CECs was associated with a better outcome (Mancuso *et al*, 2006).

Very few studies have evaluated changes in the number of CECs and CEPCs in the peripheral blood of patients receiving VEGF inhibitors. In general, CEC concentrations decrease after administration of VEGF inhibitors (Table 2a and 2b). However, this is not a consistent observation. When patients with rectal cancer were treated with bevacizumab, the concentration of CECs reduced (Willett *et al*, 2005), whereas in patients with gastrointestinal stromal tumour treated with sunitinib (a broad spectrum receptor TKi that inhibits c-kit and VEGFR), there was a transient increase in CECs that was associated with a better outcome (Norden-Zfoni *et al*, 2007).

Only a few studies have enumerated the number of CECs and CEPCs in the circulation of patients receiving VEGF inhibitors. However, significant methodological problems have to be overcome before these biomarkers can be incorporated routinely into multi-centre trials. To date, repeated pre-treatment samples have not been collected and therefore confidence intervals for individuals have not been clearly established, obscuring decisions with regard to treatment-induced effects. These problems are compounded by multiple platforms and antigen-selection suites (Mutin *et al*, 1999; Mancuso *et al*, 2001; Rowand *et al*, 2007; Dellapasqua *et al*, 2008) used to characterise CECs/CEPCs. The common technique used for analysis is immunomagnetic bead isolation or immunophenotyping using the flow cytometer. However, there is a lack of consensus regarding the choice of markers to be used. Real-time PCR using CD146 mRNA has been proposed as an alternative method for enumerating CECs (Furstenberger *et al*, 2005). At present, no markers are considered specific for CECs and a clearer antigen profile is required for isolating these cells.

In summary, the studies in Tables 2a and 2b identify a biomarker signature observed in patients treated with VEGF inhibitors. This includes an increase in VEGF (with or without VEGF-C), a decrease in VEGFR-2 (and sometimes in VEGFR-3) and, in some studies, a decrease in CECs. Occasionally, these biomarker changes have been of prognostic significance but none have been qualified as having predictive value. Although it is possible that the predictive potential of these biomarkers has not been tested in appropriately designed studies, it is important to note that many of the early studies focused on drugs with relatively high IC_50_ (e.g., semaxanib, SU5416), which therefore were less potent and perhaps less effective than the VEGFR TKis currently in the clinic, thereby reducing the chances of measuring a change in biomarker concentration on drugs that had more rapid clearance (e.g., vatalanib, PTK/ZK) or on trials that were too small to generate statistically significant results.

## Imaging biomarkers for VEGF inhibitors

Conventional radiological reporting systems for new drugs rely on one-dimensional (Response Evaluation Criteria in Solid Tumours) or two-dimensional (WHO criteria) response-assessment schemes. Neither is well suited to the assessment of anti-angiogenic agents, the principal effect of which is tumour cytostasis. Thus, early clinical trials of VEGF inhibitors sought pharmacological proof of concept by examining changes in the tumour vasculature, predominantly through the use of MRI, which is a technology that is non-invasive, sensitive and avoids ionising radiation.

Of all the biomarkers that have been tested in trials of VEGF inhibitors, the most consistent findings have been achieved with dynamic contrast-enhanced MRI (DCE-MRI), in keeping with the proposed mechanism of action of the drugs (Tables 3a and 3b). Transfer constants such as K^trans^, a composite of the vascular permeability and endothelial surface area, are reduced in patients receiving VEGF inhibitors, and multiple studies have demonstrated a dose level–response relationship. In addition, a second relationship, the correlation between the magnitude of reduction in transfer constants and the attainment of stable or better disease, has been widely reported (Morgan *et al*, 2003; Mross *et al*, 2005; Thomas *et al*, 2005; Hahn *et al*, 2008). Although many of these studies were small and confounded by inter-patient heterogeneity; generally data show that patients whose tumours undergo at least a 50% reduction in DCE-MRI parameters attain stable or better disease. Thus, DCE-MRI perhaps holds the greatest promise as a prognostic and/or predictive biomarker for VEGF inhibitors, and recent data have highlighted the potential of another DCE-MRI-derived parameter, v_p_ (the fractional plasma volume), as a further candidate biomarker that may show clinical utility in trials of VEGF inhibitors (Hahn *et al*, 2008).

Dynamic contrast-enhanced MRI is more complex than computed tomography (CT) in terms of the ease with which it can be incorporated into multi-site studies; for this reason, many centres are testing the relationship between dynamic CT and DCE-MRI. On the basis of prognostic data gathered from analysis of the tumour vasculature seen in CT scans of patients with advanced ovarian cancer treated with conventional cytotoxic therapy (O’Connor *et al*, 2007), together with ongoing comparisons between dynamic CT and DCE-MRI, it is likely that there will be an expansion in research to assess whether these techniques can serve as predictive biomarkers in patients treated with VEGF inhibitors.

Recent interest in MRI techniques that do not require contrast has highlighted blood oxygenation level-dependent (BOLD) imaging and arterial spin labelling (ASL) (de Bazelaire *et al*, 2008). Arterial spin labelling, a technique in which protons entering the zone of interest are magnetised, was developed for imaging the vasculature of the brain. Although initial results with ASL in bodies of patients treated with VEGF inhibitors show promise as a prognostic biomarker (de Bazelaire *et al*, 2008), ASL is technically challenging when used to image the body and usually requires 3-T MRI machines. Blood oxygenation level-dependent imaging, a technique that relies on the paramagnetic effects of deoxyhaemoglobin, can be used to provide information on the oxygenation status of the patient's tumour, and in particular the oxygen status in tumour vessels. However, although anatomical resolution is good, the signal-to-noise ratio is relatively low and, although this can be increased by administering carbogen, such a procedure can be unpleasant for the patient (Padhani *et al*, 2007). Arterial spin labelling and BOLD are both attractive techniques, but have not been fully evaluated as biomarkers for VEGF inhibitors and for now are likely to remain confined to specialist imaging centres.

Hypoxia is a key mediator of angiogenesis, at least in part because it induces the expression of VEGF. However, there have been very few attempts to use hypoxia-imaging strategies as biomarkers for VEGF. In addition to BOLD, which has not been used to evaluate VEGF inhibitors in the clinic, positron emission tomography (PET) imaging tracers have been used to image hypoxia in the clinic. Both ^18^F-MISO, which is retained in hypoxic cells through electron transfer that prevents egress from cells, and ^60^Cu-ATSM, which is retained through mechanisms that are unclear but depend on the redox state of cells, have been used to image hypoxia (Padhani *et al*, 2007). No studies have been reported to date on the imaging of hypoxia using these tracers in patients treated with VEGF inhibitors. The PET perfusion tracer, ^15^O-H_2_O, has also not been investigated in this setting.

The most widely used PET tracer, ^18^F-FDG, has only been evaluated in small series in patients treated with VEGF inhibitors. In patients with rectal cancer (Willett *et al*, 2004), administration of bevacizumab did not change FDG uptake over a 12-day period, despite positive pharmacological proof-of-principle studies conducted during this time period. This lack of effect has not been explained but could be due to the upregulation of the glucose transporter in hypoxic cells. Thus, if bevacizumab increases tumour cell hypoxia, paradoxically, one might observe either no change or an increase in FDG uptake.

In place of ^18^F-FDG, recent interest has focused on ^18^F-fluorothymidine (^18^F-FLT), which is incorporated into newly synthesised DNA and is taken as a surrogate for cellular proliferation. In both primary and metastatic colorectal cancer, [^18^F]FLT uptake (SUV) correlates with proliferative activity, as determined by MIB-1 immunohistochemistry (Francis *et al*, 2003). Interestingly, changes in ^18^F-FLT uptake were associated with OS in patients with malignant gliomas treated with combination of irinotecan and bevacizumab (Chen *et al*, 2007; Sohn *et al*, 2008). An important confounding factor for future studies is that effective anti-angiogenic therapy impairs blood vessel function and tumour perfusion. Unless detailed dynamic studies of tracer uptake are conducted to take this factor into account, there is a risk that SUV analysis of ^18^F-FLT in patients will overestimate the anti-proliferative effects of VEGF inhibitors. Nevertheless, this is the only PET tracer that has yielded potentially useful data with VEGF inhibitors and further exploration is warranted to determine whether it can be used as a predictive biomarker.

In summary, the most promising candidate biomarkers for VEGF inhibition arise from DCE-MRI evaluation of the effect of VEGF inhibitors in solid tumours. The relationships between dose level and MRI effect and between MRI effect and clinical benefit highlight the potential for such imaging to be evaluated as predictive biomarkers. In particular, the observation that patients whose tumours undergo a >50% reduction in contrast uptake or transfer constants in response to VEGF inhibitors usually attain stable or better disease, thus leading to the following questions regarding patient management: If we know that patients attaining 50% reduction in DCE-MRI parameters benefit from the drug, can we escalate the drug dose until this end point is reached? If dose escalation attains this degree of DCE-MRI effect, will it result in clinical benefit? Correspondingly, in patients who do not achieve 50% reduction in DCE-MRI parameters, should we stop the VEGF inhibitors?

## Tissue biomarkers for VEGF inhibitors

The majority of cancer diagnoses are achieved through light microscopic and immunohistochemical characterisation of the malignant tissue. Therefore, the availability of these blocks is a factor to be considered while looking for predictive biomarkers for VEGF inhibitors, accepting the caveat that the small proportion of tumours examined in such studies may not be representative of the whole cancer burden. For at least 10 years, multiple studies have reported the relationship between microvessel density (MVD), the product of angiogenesis and metastasis, and survival in solid tumours (Hasan *et al*, 2002). As the principal receptor implicated in driving angiogenesis was VEGFR-2, it was logical to test the value of MVD, VEGFR-2 and phospho-VEGFR2 as potential predictive biomarkers for VEGF inhibitors. Despite incorporation into multiple studies, these trials have largely been unable to confirm the hypothesis (e.g., Jubb *et al* (2006)), although some smaller studies (Yang *et al*, 2008) have reported a relationship between MVD (based on CD31) and positive response. Putative explanations for the lack of predictive value of MVD have focused on the lack of congruence between tumour metabolism, perfusion, growth and vessel density in clinical studies (Hlatky *et al*, 2002). As an alternative, various groups have used EC proliferation measured by double-staining techniques as an indicator of angiogenesis (Hillen *et al*, 2006; Wedam *et al*, 2006). Although a prognostic association was observed in melanoma, their role as a potential biomarker for VEGF inhibitors needs to be investigated further. More recent *in vivo* data have demonstrated that multiple cellular lineages, such as myeloid (Shojaei *et al*, 2007) and mesenchymal (Crawford *et al*, 2009) cells, present in and around new blood vessels can modulate sensitivity to VEGF inhibitors. Thus, more detailed cell biological studies of blood vessels in tumours may be required to determine the potential biomarker value of MVD.

Perhaps the most attractive tissue biomarker that could serve in a predictive capacity is phospho-VEGFR-2. In patients with inflammatory breast carcinoma, administration of bevacizumab resulted in significantly reduced phospho-VEGFR-2. This was coupled with a marked increase in tumour cell apoptosis, but no significant change in proliferation (Wedam *et al*, 2006). In a phase I trial of a VEGFR-2-binding di-Fab fragment, biopsy data were compatible with the proposed mechanism of action (Ton *et al*, 2007). However, such reports are very infrequent for at least two reasons: processing tissues from patients to detect phospho-proteins requires extremely rapid tissue preservation to avoid de-phosphorylation of receptors. Second, there are very few antibodies that bind with sufficient specificity to phospho-VEGFR-2. Whether a validated biomarker assay of anti-phospho-VEGFR-2 could be used successfully in a multi-site study remains to be established.

## Genetic biomarkers for VEGF inhibitors

Angiogenesis is a host-mediated phenomenon (Ferrara, 2001) in which the heterogeneous response to VEGF inhibitors may be related to inherited variations in genes coding for products that regulate angiogenesis.

Few studies have reported an association between clinical outcome and single-nucleotide polymorphisms (SNPs) in genes for VEGF. When patients with metastatic breast cancer were treated with paclitaxel and bevacizumab (E2100 trial), SNP analysis demonstrated that VEGF-2578 AA and VEGF 1154-A genotypes were associated with better OS but not response rate (RR) or PFS (Schneider *et al*, 2008). In contrast, those patients who received bevacizumab had a better RR and PFS but not OS, thereby challenging the pathophysiological role of these SNPs with regard to bevacizumab efficacy.

Multiple genes affect the efficacy of VEGF inhibitors. In patients with ovarian cancer who received cyclophosphamide and bevacizumab (Schultheis *et al*, 2008), those with an A/A or A/T genotype for Il-8 T-251A attained a lower RR than did those with a homozygous T/T genotype (*P*=0.006). The study also showed an association between PFS and polymorphisms involving CXCR2 C+785T (*P*=0.026), VEGF C+936T (*P*=0.061) and adrenomedullin dinucleotide repeat polymorphisms (*P*=0.008). Although of initial interest, these findings should be explored in further prospective trials and it will be important to define the structure–function relationships for particular variants that are associated with a better or worse prognosis.

## Hypertension as a biomarker of response

Hypertension is one of the most common toxicities in patients taking VEGF inhibitors. The Hurwitz paper reported an overall incidence of 22.4%, with 11% developing grade 3 hypertension (Hurwitz *et al*, 2004). In general, the level of hypertension is dose-level related, although the exact mechanism remains unexplained. One hypothesis is that VEGF signalling regulates nitric oxide synthase. Thus, VEGF inhibitors reduce the synthesis of nitric oxide, increasing vasoconstriction and therefore hypertension. If this is the case, then hypothetically (Maitland *et al*, 2006), an increase in blood pressure should reflect successful inhibition of the VEGF pathway. Multiple trials have corroborated this hypothesis: Of 39 patients receiving irinotecan, fluorouracil and bevacizumab for metastatic colorectal cancer, the RR and PFS (median: 14.5 *vs* 3.1 months, *P*=0.04) were significantly better for patients who had bevacizumab-induced grade 2–3 hypertension (Scartozzi *et al*, 2009). In the E2100 study on advanced breast cancer, patients who experienced grade 3 or 4 hypertension survived significantly longer (38.7 *vs* 25.3 months, *P*=0.002), although hypertension was seen in patients with VEGF634CC and VEGF1498TT genotypes (Schneider *et al*, 2008). A retrospective study involving multiple tumour types treated with axitinib, an oral VEGF inhibitor, has shown an association between diastolic blood pressure of ⩾90 mm Hg and survival (O. Rixe *et al*, 2008; Rini *et al*, 2008b).

Vascular endothelial growth factor inhibitor-induced hypertension seems to show dose level-dependent effects and therefore, as proposed for DCE-MRI, it is appropriate to ask whether we should increase the dose of VEGF inhibitors, if tolerated, until we observe hypertension.

## Future directions

The above data identify DCE-MRI, particular circulating parameters (VEGF and VEGFR2) and hypertension as candidate prognostic biomarkers for VEGF. It is now important to assess these candidates on the basis of various parameters. First, high-quality biomarker studies should be conducted to test the predictive value of these candidate biomarkers when carried out using GCLP-validated assays in optimised clinical trial designs. Second, we should test the biomarker hypothesis in a randomised trial setting, which is that dose escalation until one of these parameters is significantly perturbed will optimise treatment and lead to better outcome. If this is possible, then which of the biomarkers should be the target against which we should escalate dose? If escalation does not increase the change in biomarker, then should the drug be discontinued?

Certain biomarkers have not been evaluated in patients receiving VEGF inhibitors, the most important of which is the imaging biomarkers of hypoxia. Interesting recent pre-clinical data have highlighted the potential importance of measuring the concentration of circulating tumour cells, which depend critically on tumour circulation for intravasation, as potential biomarkers of VEGF inhibitors (Ebos *et al*, 2009; Paez-Ribes *et al*, 2009; Reynolds *et al*, 2009).

Vascular endothelial growth factor inhibitors have proven clinical value in multiple clinical settings. If we are to use these agents in the best way and, most critically, if we are to develop combination regimens that build on their efficacy, it is vital to identify who to treat using predictive biomarkers and with what dose and schedule, as determined by pharmacodynamic biomarkers. Strong biomarker research offers a realistic opportunity to address these pivotal questions.

## Figures and Tables

**Table 1:** VEGF-targeted therapies

**Mechanism of action**	**Examples of drugs**
Anti-VEGF monoclonal antibody	Bevacizumab
Small molecule receptor tyrosine kinase inhibitor (TKi)	Sunitinib
VEGF-Trap (soluble VEGF receptor)	Aflibercept

Abbreviation: VEGF=vascular endothelial growth factor.

**Table 2a:** Anti-VEGF antibodies and circulating biomarkers

**References**	**Drug, disease and trial**	**Biomarkers**	***N***	**Drug-induced changes**	**Prognostic and predictive values**
Dowlati *et al* (2008)	Carboplatin and Paclitaxel± Bevacizumab NSCLC (E4599); Phase 2/3	VEGF E-selectin FGF-2 ICAM	160	↓E-selectin ↑FGF-2	Baseline VEGF predicts response (*P*=0.01) -Low baseline VEGF: better PFS (*P*=0.04) -Low ICAM: better OS (*P*=0.00005),1 year survival and high RR (*P*=0.02)
Siegel *et al* (2008)	Bevacizumab Unresectable HCC Phase 2	VEGF SDF-1 HUVEC	8	↓VEGF and SDF-1 ↓HuVEC angiogenic score	↑VEGF and SDF-1 on progression -VEGF and SDF-1 levels correlate with angiogenic score
Yang *et al* (2003)	Bevacizumab mRCC; Phase 2	VEGF	113	↑VEGF	NS
Nimeiri *et al* (2008)	Bevacizumab+Erlotinib Recurrent ovarian cancer Phase 2	sVEGFR-2 Urine VEGF	11	NS	NS
Ko *et al* (2008)	Gemcitabine+Cisplatin+Bevacizumab Pancreatic cancer; Phase 2	VEGF FGF-2	46	↑VEGF ↑FGF-2	NS
Garcia *et al* (2008)	Cyclophosphamide+Bevacizumab Ovarian cancer; Phase 2	VEGF E-selectin TSP-1	70	↑VEGF and ↓TSP-1	NS
Denduluri *et al* (2008)	Bevacizumab Breast cancer Pilot study	VEGF sVCAM-1 sVEGFR-2	21	↑sVCAM-1 ↑sVEGFR-2	NS
Varker *et al* (2007)	Bevacizumab±IFN*α*-2b Malignant melanoma Phase 2	VEGF FGF-2	32	NS	NS
Yao *et al* (2008)	Octreotide+INF*α*-2b+ Bevacizumab NET; Phase 2	FGF-2 IL-8	36	↓FGF-2 ↑ IL-8	NS
Jayson *et al* (2005)	HuMV833 Advanced cancer Phase 1	VEGFR-1, IL-8, sVCAM-1, FGF-2, E-selectin, HGF	20	↑VEGF ↓FGF, HGF	NS
Dellapasqua *et al* (2008)	Cyclophosphamide+Capecitabine+ Bevacizumab Breast cancer; Phase 2	CEC CEPC	46	↓CEC	High baseline CECs correlate with OR (*P*=0.02), clinical benefit (*P*=0.01) and improved PFS (*P*=0.04)
Willett *et al* (2005)	Chemoradiotherapy+Bevacizumab Rectal cancer; Phase 1	CEC CEPC	6	↓CEC/CEPC	NS

Abbreviations: VEGF=vascular endothelial growth factor; CEC=circulating endothelial cell; CEPC=circulating endothelial progenitor cell; FGF-2=fibroblast growth factor-2; HCC=hepatocellular carcinoma; HGF=hepatocyte growth factor; HUVEC=human umbilical vein endothelial cell; ICAM=intercellular adhesion molecule; INF*α*-2b=interferon *α*-2b; mRCC=metastatic renal cell carcinoma; NET=neuroendocrine tumour; NS=not significant; NSCLC=non-small-cell lung cancer; OR=overall response; OS=overall survival; RR=response rate; SDF-1=stromal cell-derived factor-1; sVCAM=soluble vascular cell adhesion molecule; sVEGFR-2=soluble VEGF receptor 2; TSP-1=thrombospondin-1.

**Table 2b:** VEGF RTKi and soluble biomarkers

**References**	**Drug, disease and trial**	**Biomarkers**	***N***	**Drug-induced changes**	**Prognostic and predictive values**
Rini *et al* (2008a)	Sunitinib Bevacizumab refractory RCC Phase 2	VEGF-A, VEGF-C, sVEGFR-3 PlGF	61	↑VEGF-A and PlGF ↓VEGF-C and sVEGFR-3	Low baseline VEGF-C (*P*=0.0006) and VEGF–R-3 (*P*=0.006) associated with longer PFS and ORR
Burstein *et al* (2008)	Sunitinib Metastatic breast cancer	VEGF, sVEGFR-2, sVEGFR-3, sKIT	64	↑VEGF, ↓VEGFR-2, VEGFR-3	↓VEGFR-3 and ↑OS (*P*=0.07) ↓sKIT and ↑TTP and ↑OS (*P*=0.0194)
Bello *et al* (2006)	Sunitinib Neuroendocrine tumour	VEGF, IL-8, sVEGFR-2, sVEGFR-3	109	↑VEGF ↓VEGFR-2, ↓sVEGFR-3	↓VEGFR-3 with PR (*n*=11) ↑Il-8 with decreased tumour size
Norden-Zfoni *et al* (2007)	Sunitinib (SU11248) Imatinib-resistant GIST Phase1/2	VEGF, sVEGFR-2	73	↑VEGF ↓sVEGFR-2	NS
Azad *et al* (2008)	Sorafenib+Bevacizumab Advanced cancer Phase 1	VEGF IL-6 IL-8	28	↑VEGF No change: IL-6, IL-8	NS
Batchelor *et al* (2007)	Cediranib (AZD2171) Glioblastoma Phase 2	VEGF, PlGF, sVEGFR-1, sVEGFR-2, FGF-2, SDF-1*α*,	16	↑VEGF, ↑PlGF ↓sVEGFR-2	PD-associated variables: ↓PlGF, ↑sVEGFR2, ↑bFGF, ↑SDF-1*α*
Drevs *et al* (2007)	Cediranib (AZD2171) Advanced cancer Phase 1	sVEGFR-1 sVEGFR-2 FGF-2, PlGF,Tie-2, E-selectin, IL- 8	83	↓sVEGFR-2 ↑VEGF, ↑PlGF	NS
Rosen *et al* (2007)	AMG 706 Advanced cancer Phase 1	PlGF, VEGF, FGF-2, sVEGFR-1	71	↑PlGF ↓sVEGFR-2	↑PlGF (*P*=0.003) and ↓VEGFR-2 (*P*=0.001) associated with change in tumour size
Jonker *et al* (2007)	Brivanib (BMS-582664) Advanced cancer; Phase 1	sVEGFR-2, Collagen IV	50	↓sVEGFR-2 ↓collagen IV	NS
Heymach *et al* (2008)	Vandetanib (AZD6474) NSCLC: 3; Phase 2 trials	VEGF	251	NS	Low baseline VEGF: low risk of disease progression
Kiura *et al* (2008)	Vandetanib (AZD6474) NSCLC; Phase 2	VEGF, sVEGFR-2, Tie-2	53	↑VEGF ↓sVEGFR-2	Low baseline VEGF and TTP Low baseline VEGF in patients with PR
Yamada *et al* (2008)	E7080 Advanced cancer; Phase 1	VEGF, FGF-2 PDGF	24	↑VEGF	NS
Drevs *et al* (2005)	Vatalanib (PTK/ZK) Advanced cancer, liver mets; Phase1/2	VEGF, FGF-2, sTie-2, and E-selectin	30	↑VEGF, FGF-2	Change in VEGF correlates with outcome (*P*=0.027)
Heymach *et al* (2004)	Semaxinib (SU5416) Soft tissue sarcoma Phase 2	Urine VEGF Urine FGF-2	13	↑VEGF, FGF-2	High baseline VEGF associated with ↓OS (*P*=0.04)
Lara *et al* (2003)	IFN*α*+Semaxinib (SU5416) RCC; Phase 2	VEGF PAI-1	25	↓VEGF, ↓PAI-1	↓PAI-1 levels in patients with SD ↓baseline VEGF in pts with PD
Stopeck *et al* (2002)	Semaxinib (SU5416) Advanced cancer; Phase 1	Urine VEGF Urine FGF-2	22	↑Urine VEGF ↑Urine FGF-2	↑Urine VEGF in responders (*P*<0.05)
Fury *et al* (2007)	Semaxinib (SU5416) Head and neck cancer; Phase 2	VEGF	35	↑VEGF	NS
Peterson *et al* (2004)	Semaxinib (SU5416) Melanoma; Phase 2	VEGF	13	↑VEGF	NS
Stadler *et al* (2004)	Semaxinib (SU5416) Homone refractory prostate cancer; Phase 2	VEGF bFGF	22	NA	NS
Norden-Zfoni *et al* (2007)	Sunitinib (SU11248) Imatin-resistant GIST Phase1/2	CEC PBMC	90	↑CECs ↓PBMC	Significant clinical correlation (*P*=0.03)
Batchelor *et al* (2007)	Cediranib (AZD2171) Glioblastoma Phase 2	CEC CEPCs	16	↓CEC ↓CEPC	↑CECs with tumour progression. ↑CEPCs with relapse after drug holidays

Abbreviations: VEGF=vascular endothelial growth factor; RTKi=receptor tyrosine kinase inhibitor; CEC=circulating endothelial cell; CEPC=circulating endothelial progenitor cell; FGF-2=fibroblast growth factor-2; GIST=gastrointestinal stromal tumour; HGF=hepatocyte growth factor; RCC=renal cell carcinoma; NA=not applicable; NS=not significant; NSCLC=non-small-cell lung cancer; OR=overall response; ORR=objective response rate; OS=overall survival; PAI-1=plasminogen activator inhibitor-1; PBMC=peripheral blood mononuclear cells; PD=progressive disease; PDGF=platelet-derived growth factor; PFS=progression-free survival PlGF=placental growth factor; PR=partial response; RR=response rate; SD=stable disease; SDF-1=stromal-cell derived factor-1; sVEGFR=soluble VEGF receptor; TTP=time to progression.

**Table 3a:** Antibody-based VEGFi and imaging biomarkers

**References**	**Drug, disease and trial**	**DCE-MRI biomarkers**	***N***	**Drug-induced changes**	**Prognostic and predictive values**
Overmoyer *et al* (2004)	Docetaxel±Bevacizumab Breast cancer; Phase 2	k_ep_	26	↓k_ep_	NS
Wedam *et al* (2006)	Bevacizumab Breast cancer; Phase 2	K^trans^, v_e_	20	↓K^trans^	NS
Jayson *et al* (2005)	HuMV833 (Anti-VEGF) Advanced cancer; Phase 1	K^trans^ k_ep_ rBV	20	↓k_ep_	NS
Ton *et al* (2007)	CDP791 (Anti-VEGFR-2) Advanced Cancer; Phase 1	K^trans^	31	No DCE-MRI change Dose-related volumetric change	NS

Abbreviations: VEGF=vascular endothelial growth factor; DCE-MRI=dynamic contrast-enhanced magnetic resonance imaging; k_ep_=rate constant; K^trans^=bi-directional transfer coefficient; rBV=regional blood volume; v_e_=volume of the extravascular extracellular space (EES).

**Table 3b:** VEGFR, TKi and imaging biomarkers

**References**	**Drug and trial**	**DCE-MRI biomarkers**	***N***	**Drug-induced changes**	**Prognostic/predictive value**
Flaherty *et al* (2008)	Sorafenib Renal cancer; Phase 2	K^trans^	17	↓K^trans^	High baseline (*P*=0.02) and % change in K^trans^ (*P*=0.01) predict PFS
Hahn *et al* (2008)	Sorafenib Renal Cancer; Phase 2	IAUC, v_p_, K^trans^	56	↓IAUC, v_p_, K^trans^	High baseline K^trans^: better PFS (*P*=0.027)
Batchelor *et al* (2007)	Cediranib (AZD2171) Glioblastoma; Phase 2	K^trans^, v_e_, vessel size	16	↓K^trans^ ↓vessel size	NS
Drevs *et al* (2007)	Cediranib (AZD2171) Advanced cancer; Phase 1	IAUC	24	↓IAUC	NS
Miller *et al* (2005)	Vandetanib (AZD6474) Breast cancer; Phase 2	IAUC, K^trans^	11	↓IAUC K^trans^	NS
Rosen *et al* (2007)	AMG706 Advanced Cancer; Phase 1	IAUC	18	↓IAUC	NS
Jonker *et al* (2007)	Brivanib (BMS-582664) Advanced cancer; Phase 1	IAUC, K^trans^	50	↓K^trans^, ↓IAUC	NS
Padhani *et al* (2006)	BIBF1120 Advanced cancer; Phase 1	IAUC, K^trans^, K_ep_	35	No consistent effect	NS
Mross *et al* (2005b)	BIBF1120 Advanced Cancer; Phase 1	IAUC, Ki	27	↓IAUC, Ki	NS
Liu *et al* (2005)	Axitinib (AG013736) Advanced cancer; Phase 1	IAUC, K^trans^	17	↓IAUC, K^trans^	NS
Mross *et al* (2005)	Vatalanib (PTK/ZK) Colorectal/breast ca Phase 1	Ki	27	↓Ki	Change in Ki: RR and progression
Thomas *et al* (2005)	Vatalanib (PTK/ZK) Advanced cancer; Phase 1	Ki	43	↓Ki	Change in Ki: predicts progression
Conrad *et al* (2004)	Vatalanib (PTK/ZK) Glioblastoma; Phase 1/2	Ki, RBV	14	↓Ki	Change in Ki predicts progression
Morgan *et al* (2003)	Vatalanib (PTK/ZK) Colorectal cancer; Phase 1	Ki	26	↓Ki	Change in Ki predicts RR and progression
O’Donnell *et al* (2005)	Semaxinib (SU5416) Advanced Cancer; Phase 1	K^trans^	24	NS	NS
Dowlati *et al* (2005)	Semaxinib (SU5416) Advanced cancer; Phase 1	Kep	11	NS	NS
Medved *et al* (2004)	Semaxinib (SU5416)	IAUC	19	↓IAUC	NS
Xiong *et al* (2004)	SU6668 Advanced cancer; Phase 1	IAUC, gradient	27	NS	NS

Abbreviations: VEGF=vascular endothelial growth factor; DCE-MRI=dynamic contrast-enhanced magnetic resonance imaging; IAUC=initial area under the contrast agent concentration – time curve; K_i_=unidirectional influx constant; k_ep_=rate constant; k^trans^=bi-directional transfer coefficient; NS=not significant; rBV=regional blood volume; RR=response rate; v_e_=volume of the extravascular extracellular space; v_p_=blood plasma volume.
